# Patterns of Daily Duration and Frequency of Breastfeeding among Exclusively Breastfed Infants in Shiraz, Iran, A 6-Month Follow-up Study Using Bayesian Generalized Linear Mixed Models

**DOI:** 10.5539/gjhs.v5n2p123

**Published:** 2012-12-19

**Authors:** Azadeh Saki, Mohammad Reza Eshraghian, Hamed Tabesh

**Affiliations:** 1 Department of Biostatistics and Epidemiology, School of Public Health, Ahvaz Jundishapur University of Medical Sciences, Ahvaz, Iran; 2 Department of Epidemiology and Biostatistics, School of Public Health and Institute of Public Health Research, Tehran University of Medical Sciences, Tehran, Iran

**Keywords:** exclusive breastfeeding, frequency of suckling, duration of suckling, breastfeeding pattern, Bayesian, Generalized Linear Mixed Model

## Abstract

**Introduction:**

Despite numerous studies on the benefits of exclusive breastfeeding during the first half year of life, little information is available on actual breastfeeding practices in terms of daily duration and frequency of suckling. This study proposes to determine daily breastfeeding patterns among exclusively breastfed infants from birth to six months.

**Subject and Methods:**

An observational prospective follow-up study of daily feeding practices among exclusively breastfed infants was conducted in 2007/2008. Mothers were recruited and interviewed during their first month postpartum health center visit. A total of 287 mothers were recruited into the study. Primary outcome measures were suckling duration and frequency of breastfeeding during daytime and nighttime. Mothers were asked at each healthcare visit to report the daily duration in minutes and the daily number of breastfeeding sessions. Mixed models were used to determine breastfeeding patterns and predictors.

**Results:**

Of 287 mothers selected for this study, 174 (61%) exclusively breastfeeding until six months after delivery. Mixed modeling showed that as the infant's age increased duration of one suckling, cumulative duration and frequency of breastfeeding during daytime, nighttime and a twenty four hour period all gradually decreased. Infants gender and receiving professional advice about breastfeeding were also significant factors in breastfeeding patterns (p<0.05).

**Conclusions:**

The one suckling duration and frequency of feeds in this study population were considerably higher than values reported in other populations. The variation of feeding patterns between exclusively breastfed infants was very wide. The distributions of one suckling duration, frequency of breastfeeding and cumulative duration of feeds were right-skewed. The current professional advices about breastfeeding are not appropriate because they do not consider unique condition within specific populations.

## 1. Introduction

The variation in breastfeeding patterns is very wide within and between populations. These patterns are influenced by maternal and infant factors and the interactions between mothers and their infants (Kramer & Kakuma, 2012; Shealy, Scanlon, Labiner-Wolfe, Fein, & Grummer-Strawn, 2008; Ghosh, Mascie-Taylor, & Rosetta, 2006; Glushuddin & Kabir, 2004; Mahgoub, Bandeke, & Nnyepi, 2002; Hornell, Aarts, Kylberg, Hofvander, & Gebre-Medlin, 1999; Chhabra, Grover, Aggarwal, & Dubey, 1998; Marriott, 1998). Previous studies have shown that the mother's ability to provide milk and the infant's demand are significant factors for the daily frequency of breastfeeding (Daly & Hartmann, 1995). Studies have also reported that the total duration of breastfeeding is related to many factors, including breastfeeding support, maternal age, education, socioeconomic status, and smoking, the infant's use of a pacifier, and co-sleeping (Olang, Heidarzadeh, Strandvik, & Yngve, 2012; Saki, Eshraghian, Mohammad, Foroushani, & Bordbar, 2010; Aarts, Hornell, Kylberg, Hofvander, & Gebre-Medlin, 1999; Perez-Escamilla et al., 1995; Ford et al., 1994).

In 2001, the World Health Organization (WHO) recommended exclusive breastfeeding from birth to six months. Exclusive breastfeeding during the first six months of infant life is found to reduce the incidence of infant diarrhea and respiratory diseases as well as infant mortality and morbidity rates, particularly in developing countries (Koyanagi et al., 2009; Quigley, Kelly, & Sacker, 2007).

Despite numerous studies highlighting the advantages of exclusive breastfeeding during the first six months of infant life (Kramer & Kakuma, 2012; Saki et al., 2010; World Health Organization [WHO], 1991), there is little data on the daily variations in breastfeeding practices and patterns in terms of daily frequency (number of feeds) and cumulative suckling duration (minutes) among exclusively breastfed infants. Instead, most previous studies tends to describe a related outcome such as breastfeeding intensity (the number of breast milk feeds (on average in 24 hours) divided by the total number of all liquid feeds (on average in 24 hours)). Information about the daily variation in frequency and duration of breast feeding can provide insights into the mechanisms of breastfeeding. This data is also essential for individualized counseling about breastfeeding within different population.

The first aim of this study is to determine breastfeeding patterns by focusing on three components: i.e. one suckling duration (minutes), frequency of feeds, and the cumulative duration of feeds separately during daytime, nighttime and twenty four hour period. This information will be gathered for exclusively breastfed infants during their first six months of life, using strict WHO criteria for exclusive breastfeeding (WHO, 1991). The second purpose is to test whether maternal factors; mother's age, education, and number of live births, receiving professional advice about breastfeeding and infant's age and gender were associated with these tree components.

## 2. Methods

### 2.1 Sampling and Participants

This was a prospective follow-up observational study. Mothers were recruited into the study from July 10, 2007 to September 10, 2007 from Shiraz healthcare centers during their first month postpartum visit. The mother-infant pairs were followed up until six months after delivery. The healthcare centers were selected using cluster sampling scheme, with-each healthcare center considered a cluster and the proportion of participants from each cluster being proportional to size of that healthcare center.

Inclusion criteria for mother-infant pairs included: mothers being residents of Shiraz, intention to breastfeed their infants for at least 6 months, infant term, singleton births, birth weight ≥ 2500 g, and infant had no medical complications. All mother-infant pairs needed to visit healthcare centers six times within six months after delivery. But some of them not presented in the healthcare centers at one or more occasions.

Exclusion criteria included infant hospitalization, feeding anything except for breast milk before six months after delivery, and unknown status of breastfeeding at the end of six months. Among 287 mother-infant pairs included in the study 174 (61%) had all conditions and were still exclusively breastfeeding at 6 months after delivery.

Seven structured interviews were developed by the researchers; the first interview, completed at the time of recruitment to the study by the nurse in healthcare centers, included maternal and neonatal demographics and background data [infant's gender, birth weight and length (according to hospital records); mother's age, education, and number of live births]. The other six interviews were similar and were used to collect infant assessment data [anthropometric measures and health status], current feeding patterns [breastfeeding exclusively (“Have you fed your baby anything except the mother's milk?”), one suckling duration (minutes) in day time and night time, and frequency of feeds in daytime and nighttime during the past 24 hours]. Mothers were also asked if they had received professional advice about breastfeeding at maternal hospitals or healthcare centers (“Have you received advice about breastfeeding until now?”). Breastfeeding counseling, according to WHO guidelines in Iran is an on-going task and is performed in some maternity hospitals and healthcare centers. Therefore, some of the mothers in this study were professionally advised about breastfeeding at the time of admission to the study. It was possible for other mothers to receive professional advice during the study, so mothers were asked about receiving professional advice about breastfeeding counseling in healthcare centers at all interviews. As a result, the number of counseled mothers could increase during the study at different follow up times.

The first study limitation was a number of loss to follow-up of mothers (among 287 women included in the study, 230 (80%) were still participating at 6 months following delivery. The second limitation was in data recording; mothers were asked to recall the approximate daily frequency and duration of breastfeeding within the last 24 hours. The mother's recordings were in agreements with their interviews. Intra class correlation of recording data of mother responses for frequency of feeds and cumulative duration of feeds during twenty four hour period were 0.84 and 0.85, respectively. So, the reliability of the data was reasonable. Inter-rater reliability among the nurses was checked by the inspectors of the Deputy for Health at Shiraz University of Medical Sciences.

### 2.2 Statistical Analysis

Linear Mixed model (LMM) and Generalized Linear Mixed Model (GLMM) were used to determine factors associated with the duration and frequency of breastfeeding, respectively. Mixed models are widely used in the analysis of clustered data, such as repeated measurements from the same mother. A GLMM enables the accommodation of none normally distributed responses and the specification of a possible nonlinear link function between the mean of the response and the predictors, and can model correlation by incorporating random effects. It is often a reasonable approximation assumed that the random effect terms have a multivariate normal distribution whose variance components are to be estimated from the data (Brown & Prescott, 2006; Zeger & Karim, 1991).

With repeated measure responses, generalized linear mixed models have been widely used to account for dependence within subjects. The GLMM has the general form:


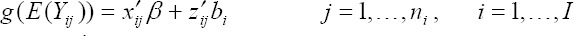


Where *y_ij_* is the response for the *j^th^* observation for the subject i; *x_ij_* is a *px1* vector of covariates associated with that response; β is the vector of regression parameters that are of scientific interest; *z_ij_* is a *qx1* subset of *x_ij_* with random coefficients; b_i_ is a *qx1* vector of random effects assumed to follow a Gaussian distribution with mean 0 and unknown variance *Σ* and g( ) are known link functions that link the expected mean of the responses to the linear combination of the predictors.

For the responses following a Gaussian distribution (such as the duration of breastfeeding), link function (g) is identity so, 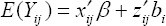 and the model is Linear Mixed Model (LMM).

When the outcomes come in the form of Poisson counts (such as frequency of breastfeeding), to obtain a linear combination the log-link function, 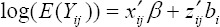 is used. So the model named as log-linear mixed model or generally, generalized linear mixed model (GLMM).

The Chi-Square test shows that the frequency of breastfeeding had a truncated Poisson distribution. This means that the minimum observed value for this variable is k (where k is greater than 0) instead of 0 at all occasions. As a result, classical log-likelihood analysis based on their joint marginal distribution was very complicated and the usual numerical method could not calculate these equations. On the other hand assuming complete Poisson distribution is incorrect and it might lead to a prediction out of the possible range of the variable.

In this study the Bayesian approach was used to accommodate this problem in fitting a GLMM to the frequency of breastfeeding and asses its dependence with different covariates.

The Bayesian approaches avoid the need for numerical integration by taking repeated samples from posterior distributions using the Gibbs sampling technique. An alternative feature of the Bayesian approach is its flexibility for full assessment of the uncertainty in the estimated random effects and functions of model parameters. Bayesian inference is carried out conditional on the observed data and does not rely on the assumption that hypothetical infinite populations of data exists. These inferences give certain advantages to Bayesian methods, such as all inferences being exact and not approximated and that the results are interpretable (Ntzoufras, 2009; Chen, 2009; Congdon, 2006).

To fit LMM for duration of breastfeeding and GLMM with Bayesian approach for frequency of breastfeeding the “nlme” package and “rjags” package in the R program were used.

### 2.3 Ethics

Ethical approval was obtained from the Ethics in Research Committee at the Deputy of Research of Tehran University of Medical Sciences. There were no anticipated physical, social or legal risks associated with participation. Informed consent was implied if participants completed the first questionnaire. It is standard practice in Iranian healthcare centers to ask participants to complete questionnaires at their health checks without written consent.

## 3. Results

Of 287 mothers recruited into this study, 174 (61%) exclusively breastfeed until 6 months after delivery (Table 1). Half of the mothers were between 25-35 years of age and only 6 (3.4%) mothers were older than 35 years. Fifty-four percent of the mothers were primiparous.

**Table 1:** Mean and standard deviation (SD) of infant age, number of mother-infant pairs at each follow up time and loss due to follow up

Occasion	Infant Age (days) Mean (SD)	Participating mothers N (%)	Drop-out mothers N (%)
1	14.4 (13.2)	287	0
2	33.4 (21.7)	283	4
3	55.5 (29.6)	279	4
4	87.9 (31.2)	271	8
5	138.9 (24.9)	257	14
6	183.6 (190)	230	27

The majority of the women in this study (74.7%) had a secondary school education (Table 2). Given that only 2 (1.1%) mothers were smokers; this factor was not included in the modeling. The proportion of the mothers who received professional advice about breastfeeding was 66.1% at the start of the study, but during the study this proportion increased and reached close to 80% (Table 2).

**Table 2:** Participants characteristics (N=174)

Variables	N (%)
Infant Gender	
Male	99 (56.9)
Female	75 (43.1)
Mother Age	
<25	81 (46.6)
25-35 years	87 (50.0)
36+	6 (3.4)
Mother Education	
Primary*	29 (16.6)
Secondary**	130 (74.7)
University	15 (8.6)
Maternal Smoking	2 (1.1)
Number of live births***	
1	95 (54.6)
2+	79 (45.4)
Professional advice about breastfeeding	
Advised mothers at admission	115(66.1)
Cumulative advised mothers within 6 month	137 (78.7)

* Primary: 1-8 years of formal education

** Secondary: 9-12 years of formal education

*** Included current child

Breastfeeding Patterns: The patterns of breastfeeding were considered in three components; frequency of feeds (numbers), single suckling duration (minutes), and the cumulative duration of feeds (minutes) separately during daytime, nighttime and twenty four hour period.

**Figure 1:**
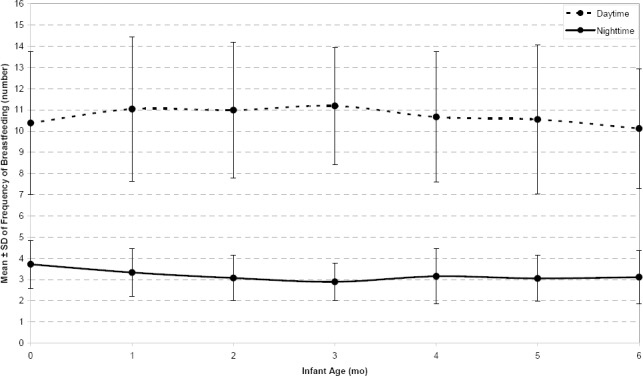
The mean ± standard deviation of frequency of breastfeeds during daytime and night time at different infant ages

Frequency of feeds: The distribution of frequency of feeds during daytime and nighttime showed in figure 1, respectively. The Kolmogorov-Smirnov test showed that the frequency of feeds followed Poisson distribution. The generalized linear mixed model with log-link function was therefore used for analyzing the frequency of breastfeeding during daytime, nighttime and twenty four hour period.

Bayesian analysis for fitting GLMM showed that the mothers’ age, education and number of live births were not significant factors for the frequency of feeds during both daytime and nighttime, as well as over a twenty four hour period. Infant age and receiving professional advice about breastfeeding were significantly associated with frequency of breastfeeding in the daytime and twenty four hour period, but receiving professional advice about breastfeeding was not a significant factor in the frequency of feeds at nighttime. Frequency of feeds was not significantly different between boys and girls in the daytime and the nighttime separately, but total frequency of breastfeeding during the twenty four hour period among boys was significantly higher than in girls. Table 3 shows the parameters estimation and 99% credible interval for parameter estimation for factors that included in the models for frequency of breastfeeding during daytime, nighttime and 24-hr period.

**Table 3:** Association of breastfeeding frequency at daytime, nighttime, and te twenty four hour period with predictors (fitted GLMM model)

Variables	Daytime			Nighttime			24-hr period		

	β	L_95%_	U_95%_	β	L_95%_	U_95%_	Β	L_95%_	U_95%_
Intercept	2.35*	2.27	2.40	1.15*	0.01	1.37	2.60*	2.56	2.68
Infant age (month)	-0.02*	-0.03	-0.01	-0.03*	-0.04	-0.01	-0.02*	-0.03	-0.01
Infant gender 0=Girl / 1=Boy	-0.08	-0.13	.006	-0.00	-0.09	0.58	-0.06*	-0.14	-0.01
RPABF** 0=No/ 1=Yes	0.14*	0.04	.165	0.01	-0.01	0.07	0.10*	0.04	0.14
N. of live births 1=“1”/ 2=“2+”	-0.02	-0.07	0.10	0.00	-0.07	0.06	0.07	-0.04	0.05
Mother education	0.04	-0.03	0.09	0.05	-0.04	0.12	-0.00	-0.04	0.05
Mother age	-0.05	-0.11	0.01	-0.04	-0.09	0.05	-0.09	-0.08	0.02

* Significant parameters which remained in the model.

** If this interval included 0, its related factor is not significant at α=.05

*** RPABF: Receiving Professional Advice about Breastfeeding

One suckling duration: The distributions of one suckling duration during both daytime and nighttime in these exclusively breastfed infants was skewed at each age. The Kolmogrov-Smirnov test showed that these variables were not normally distributed, the third root transformation was used to achieve the normality assumption for fitting linear mixed models on one suckling duration separately during daytime and nighttime.

The results of LMM showed that except for infant age and receiving professional advice about breastfeeding the other factors were not significantly associated with suckling duration during daytime. Analyses on suckling duration during the nighttime showed that in addition to infant age and receiving professional advice about breastfeeding, the number of live births had a significant effect on suckling duration during nighttime (Table 4).

**Table 4:** Association of suckling duration of breastfeeding at daytime, nighttime, and te twenty four hour period with predictors (fitted LMM model)

Variables	Daytime			Nighttime		

	Β	S.E	P	Β	S.E	P
Intercept	2.42*	0.15	0.000	2.06*	0.15	0.000
Infant age (month)	-0.02*	0.00	0.000	-0.03*	0.00	0.000
Infant gender 0=Girl / 1=Boy	0.09	0.10	0.379	-0.07	0.10	0.465
RPABF** 0=No/ 1=Yes	0.21*	0.10	0.045	0.40*	0.11	0.000
N. of live births	0.16	0.09	0.084	0.23*	0.09	0.015
Mother education 1=Primary/ 2=Secondary / 3=University	-0.02	0.10	0.826	-0.05	0.10	0.631
Mother age 1= “<25”/ 2= “25-35” / 3= “>35”	0.03	0.04	0.347	0.05	0.04	0.187
RPABF * N. of live births	-0.19*	0.07	0.007	-0.25	0.07	0.000

* Significant parameters which remained in the model

** RPABF: Receiving Professional Advice about Breastfeeding

Comparing the mean of suckling duration during daytime and nighttime showed that the one suckling duration during nighttime was significantly shorter than that during daytime at different infant ages (Table 5).

**Table 5:** Comparing the mean of suckling duration during daytime and night time at different infant ages

Infant age (days)	One suckling duration (minutes) during:				Paired Correlation	P-value from Paired Sample t-test

	Daytime		Nighttime			

	Mean	SD	Mean	SD		
1-14	17.6	9.3	15.6	6.8	.691*	.001
15-29	16.7	6.5	15.0	7.5	.583*	.010
30-59	16.9	6.4	14.5	8.1	.474*	.000
60-89	16.5	6.6	14.5	7.3	.584*	.000
90-119	17.4	6.2	12.6	3.9	.320*	.000
120-149	15.0	6.7	12.5	5.7	.583*	.000
150-179	16.9	6.8	11.8	5.0	.256*	.000
180-189	15.3	6.4	11.9	5.1	.478*	.000

* significant correlation with P-value < 0.01

**Figure 2:**
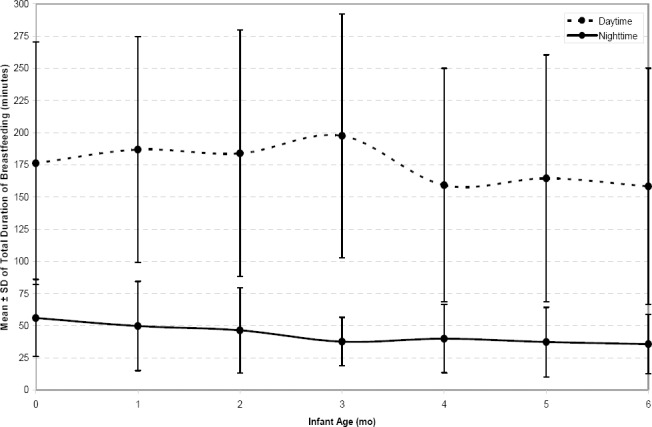
The mean ± standard deviation of total duration of breastfeeds during daytime and night time at different infant ages

Cumulative suckling duration: The error bar plot of cumulative suckling duration showed in Figure 2. The Kolmogorov-Smirnov test showed that the distribution of cumulative suckling duration during a twenty four hour period was not normal. The third root transformation was used to normalize this variable and performing LMM.

Results from LMM show that infant age and gender, number of live births and receiving professional advice about breastfeeding were significant factors for cumulative suckling duration during daytime and at the twenty four hour period. However, in relation to the cumulative duration of feeds during nighttime, the number of live births and infant's gender were not significant factors. Table 6 shows the parameters estimation, standard errors and p-values of all factors that are included in the models for the cumulative suckling duration in daytime, nighttime and the twenty four hour period.

**Table 6:** Association between cumulative suckling duration of breastfeeding at daytime, nighttime, and the twenty four hour period with predictors (fitted LMM model)

Variables	Daytime			Nighttime			24-hr period		

	β	S.E	P	β	S.E	P	β	S.E	P
Intercept	4.02*	0.64	0.000	3.35*	0.29	0.000	4.49*	0.62	0.000
Infant age (month)	-0.07*	0.01	0.000	-0.07*	0.01	0.000	-0.09*	0.01	0.000
Infant gender 0=Girl / 1=Boy	0.70*	0.27	0.010	-0.02	0.19	0.896	0.63*	0.26	0.016
RPABF** 0=No/ 1=Yes	1.36*	0.47	0.004	0.57*	0.21	0.008	1.40*	0.46	0.002
N. of live births 1="1"/ 2="2+" (1=Prima, 2=Multi)	0.63*	0.27	0.021	0.19	0.18	0.313	0.66*	0.26	0.011
Mother education	-0.25	0.26	0.342	-0.12	0.19	0.522	-0.28	0.26	0.280
Mother age 1= “<25”/ 2= “25-35” / 3= “>35”	0.02	0.11	0.857	0.11	0.07	0.096	-0.06	0.10	0.542

* Significant parameters which remained in the model.

** RPABF: Receiving Professional Advice about Breastfeeding

Frequently breastfeeding: The frequency of feeds more than 18 times, that also indicated extreme values for cumulative duration of feeds, during twenty four hour were considered as extreme values (frequently breastfed). The number of frequently breastfed infants and their weight gain at each ages presented in Table 7. Analyzing on extreme values showed that the weight gain (g/day) of infants who fed most frequently was similar to infants who fed 6-18 times during the twenty four hour period (Table 7).

**Table 7:** Comparing infant’s weight gain (g/day) between infants who fed > 18 times and those who fed 6-18 times during the twenty four hour period

Infants Age (days)	Frequency of Feeds During 24-hr Period						P-value

	6-18 feeds			>18 feeds			

	N	Mean	SD	N	Mean	SD	
1-14	128	14.1	39.9	9	18.1	31.6	0.771
15-29	78	30.4	16.1	20	33.7	19.0	0.431
30-59	153	35.4	16.1	16	39.0	13.0	0.446
60-89	138	34.5	9.1	14	33.3	5.8	0.606
90-119	62	29.3	8.1	6	26.6	6.9	0.430
120-149	162	28.1	5.9	7	28.7	6.0	0.813
150-179	70	24.8	4.8	3	25.3	2.7	0.847
180-189	129	24.1	3.8	7	23.4	3.5	0.613

## 4. Discussion

Several studies have stressed the importance of exclusive breastfeeding during the first six months of infant life and its social, economic and health benefits. Few studies have investigated daily breastfeeding practices and patterns among exclusively breastfed infants. The present study was conducted to provide information of value for the understanding of breastfeeding patterns of exclusively breastfed infants during the first six months of infant life.

The descriptive findings showed that the average of both frequencies and cumulative duration of feeds in a twenty four hour period among exclusively breastfed infants in this study population were higher than other populations (Shealy et al., 2008; Ghosh et al., 2006; Hornell et al., 1999). Two similar longitudinal descriptive studies in Sweden and US, two industrialized countries, reported that the median cumulative suckling duration in a twenty four hour period was approximately 120 minutes at first month and 80 minutes at six month, and the median frequencies of feeds showed a declining trend from 8 feeds at first month to 7 at six months (Shealy et al., 2008; Hornell et al., 1999). While, in the present population, the median of cumulative suckling duration was 228 minutes at one month and decreased to 175 minutes at six months. Also the median of frequencies of feeds was 14 times at 1 mo and 13 times at 6 mo of infant's age.

Another longitudinal study on breastfeeding pattern during an eight hour daytime work period among rural Bangladeshi woman (extremely poor population) showed that the mean of cumulative suckling duration was 68 minutes at age < 60 days and 35 minutes at age between 120 and 180 days, and the frequencies of feeds was 8 feeds at age < 60 days and decreased to 5 feeds at age 120-180 days (Ghosh et al., 2006). In compare to our study the mean of cumulative duration of feeds during the16-hr daytime period was 186 min at 1 mo and decrease to 156 min at 6 mo, and the mean of frequencies of feeds was 17 feeds at 1 mo and decreased to 15 feeds at 6 mo.

The other important finding was that the distributions of frequency and duration of feeds during the twenty four hour period were positively skewed, meaning that some extreme values exist among observations. The frequency of feeds more than 18 times during twenty four hour were considered as extreme values. Analyzing on extreme values showed that the weight gain (g/day) of infants who fed most frequently was similar to infants who fed 6-18 times during the twenty four hour period (Table 7). As a result, the extremely number of feeds could not increase the infant weight gain/milk intake (Albernaz, Victora, Haisma, Wright, & Coward, 2003).

These findings supported that the most times of these breastfeeding's were not necessary and the mothers could not diagnose the real demand of their infants. As an additional result is this hypothesis that frequently breastfeeding among these infants was due to their dependency on mothers’ breast and it seems that for these infants the mothers’ breast was akin to a pacifier.

However the frequency and cumulative duration of feeds in this population were high, the strongly significant effect of professional advice about breastfeeding on increasing both duration and frequency of feeds was observed.

Since too frequent breastfeeding may leads to short and temporal satisfaction and definite negative effect on taking solids when the complementary feeding began (Perera, Fernando, Warnakulasuria, Ranathunga, 2011), the effect of professional advice about breastfeeding on extreme observations was investigated and found that the rate of the extreme observations among mothers who received professional advice about breastfeeding was similar to other mothers. It means that however the professional advice affected the breastfeeding pattern but its effect was only increasing the number and duration of feeds for all mothers and it could not modify the daily breastfeeding pattern.

As shown in Tables 5, 6 and 8, one suckling duration, frequency of feeds and cumulative duration of feeds during a twenty four hour period decreased gradually with increasing age of the infant. Previous studies supported these findings (Shealy et al., 2008; Ghosh et al., 2006; Hornell et al., 1999).

The observed higher frequency and cumulative duration of feeds among boys (although not significant for suckling duration) found in previous studies in developing countries (Ghosh et al., 2006; Hornell et al., 1999).

Mother's age and education were additional factors that influenced the relationship to the breastfeeding patterns. These factors were not significantly related to frequency and duration of breastfeeding in the present study.

## 5. Conclusion

Compared to the few studies conducted in developing countries, both frequency and cumulative duration of breastfeeding in this population was considerably high. The distributions of frequency and cumulative duration of feeds were skewed, showing extreme values exist among observations. Analyses on extreme values show that the weight gain of infants who feed extremely (more than 18 times during a twenty four hour period) was similar to infants who feed 6-18 times. We also found that professional breastfeeding counseling was associated with decreased rates of the extreme observations of breastfeeds. The current daily breastfeeding patterns are not suitable and the professional advices are not appropriate. The advices about breastfeeding are in direction of increasing the frequency and duration of feeds and it is not suitable for all mothers.

The result of this study shows that the breastfeeding pattern varies between populations and individuals, but that professional advice about breastfeeding could affect the pattern of breastfeeding. In-depth in prospective follow-up studies investigating breastfeeding patterns are needed to find the related psychological, cultural, and socio-economic factors that influence the daily pattern of the breastfeeding and determine the optimum and critical values for one suckling duration and frequency of feeds. Also a follow up study after starting complementary feeding among extremely breastfed infants to find the long time effect of this nutrition habit was recommended. This information is necessary for adjusting breastfeeding counseling for populations and individuals.
